# Fecal calprotectin is a useful biomarker for predicting the clinical outcome of granulocyte and monocyte adsorptive apheresis in ulcerative colitis patients: a prospective observation study

**DOI:** 10.1186/s12876-021-01889-0

**Published:** 2021-08-06

**Authors:** Nobuhiro Ueno, Yuya Sugiyama, Yu Kobayashi, Yuki Murakami, Takuya Iwama, Takahiro Sasaki, Takehito Kunogi, Keitaro Takahashi, Kazuyuki Tanaka, Katsuyoshi Ando, Shin Kashima, Yuhei Inaba, Kentaro Moriichi, Hiroki Tanabe, Masaki Taruishi, Yusuke Saitoh, Toshikatsu Okumura, Mikihiro Fujiya

**Affiliations:** 1 Division of Metabolism and Biosystemic Science, Gastroenterology, and Hematology/Oncology, Department of Medicine, Asahikawa Medical University, Midorigaoka-Higashi 2-1-1-1, Asahikawa, Hokkaido 078-8510 Japan; 2 Asahikawa Kosei General Hospital, Asahikawa, Hokkaido Japan; 3 Asahikawa City Hospital, Asahikawa, Hokkaido Japan

**Keywords:** Fecal calprotectin, Ulcerative colitis, Biomarker, Granulocyte/Monocyte Apheresis (GMA), Endoscopic remission, Clinical remission, Colonoscopy

## Abstract

**Background:**

Granulocyte and monocyte adsorptive apheresis (GMA) is widely used as a remission induction therapy for active ulcerative colitis (UC) patients. However, there are no available biomarkers for predicting the clinical outcome of GMA. We investigated the utility of Fecal calprotectin (FC) as a biomarker for predicting the clinical outcome during GMA therapy in active UC patients.

**Methods:**

In this multicenter prospective observation study, all patients received 10 sessions of GMA, twice a week, for 5 consecutive weeks. FC was measured at entry, one week, two weeks, and at the end of GMA. Colonoscopy was performed at entry and after GMA. The clinical activity was assessed based on the partial Mayo score when FC was measured. Clinical remission (CR) was defined as a partial Mayo score of ≤ 2 and endoscopic remission (ER) was defined as Mayo endoscopic subscore of either 0 or 1. We analyzed the relationships between the clinical outcome (CR and ER) and the change in FC concentration.

**Result:**

Twenty-six patients were included in this study. The overall CR and ER rates were 50.0% and 19.2%, respectively. After GMA, the median FC concentration in patients with ER was significantly lower than that in patients without ER (469 mg/kg vs. 3107 mg/kg, p = 0.03). When the cut-off value of FC concentration was set at 1150 mg/kg for assessing ER after GMA, the sensitivity and specificity were 0.8 and 0.81, respectively. The FC concentration had significantly decreased by one week. An ROC analysis demonstrated that the reduction rate of FC (ΔFC) at 1 week was the most accurate predictor of CR at the end of GMA (AUC = 0.852, *P* = 0.002). When the cut-off value of ΔFC was set at ≤ 40% at 1 week for predicting CR at the end of GMA, the sensitivity and specificity were 76.9% and 84.6%, respectively.

**Conclusion:**

We evaluated the utility of FC as a biomarker for assessing ER after GMA and predicting CR in the early phase during GMA in patients with active UC. Our findings will benefit patients with active UC by allowing them to avoid unnecessary invasive procedures and will help establish new strategies for GMA.

## Introduction

Ulcerative colitis (UC) is a chronic intestinal disorder of unknown etiology and characterized by a relapsing and remitting course [1, 2]. The diagnosis and assessment of the disease activity has been based on clinical symptoms, laboratory measurements, findings of endoscopy and pathological examinations [1]. Therefore, the development of a non-invasive and simple biomarker for evaluating the disease activity is considered necessary for the clinical management of UC.

Calprotectin is a complex of the mammalian proteins S100A8 and S100A9 found in the cytosol of human neutrophils, monocytes and macrophages [3]. It is released into the intestinal lumen by cellular degranulation of neutrophils in response to intestinal mucosal inflammation; thus, the fecal calprotectin (FC) directly reflects the migration of neutrophils into the intestinal mucosa in inflammatory bowel diseases (IBD) [4]. Calprotectin is very stable and resistant to degradation and can be detected in stool for three days when left at room temperature [5, 6]. Previous studies have reported that the FC concentration is well correlated with endoscopic severity [7, 8]. and histological findings [9] and can also predict clinical relapse during maintenance therapy for UC [10, 11]. Thus, in the clinical setting, FC is widely used as a non-invasive biomarker for monitoring intestinal inflammation in UC. Several studies have further explored its value as a biomarker for predicting and assessing the clinical response to induction therapy in patients with active UC [12–16]. However, the utility of the FC concentration as a biomarker for predicting the clinical outcome during remission induction therapy has not been established.

Granulocyte and monocyte adsorptive apheresis (GMA) with an Adacolumn (JIMRO Co., Takasaki, Japan) has been applied as a non-pharmacological treatment strategy. GMA is widely used as a remission induction therapy for patients with active UC in Japan and is also available in the European Union. The mechanism underlying GMA involves the Adacolumn which is filled with cellulose acetate carrier beads, interacting with fragment crystallizable-gamma receptor (FcγR) expressed at the surface of leukocytes and selectively adsorbing granulocytes, monocytes/macrophages, a significant fraction of platelets and a small number of lymphocytes from circulation [17, 18]. Numerous reports have described the clinical efficacy and safety of GMA in patients with active UC [19–28]. However, it remains difficult to evaluate the clinical efficacy of GMA, as there are no available biomarkers for assessing and predicting the clinical response.

We hypothesized that FC might be a useful biomarker for evaluating the clinical efficacy of GMA, as GMA with an Adacolumn selectively adsorbs granulocytes, including neutrophils, which are rich in calprotectin. Very few clinical studies have found FC to be useful as a biomarker for GMA, and its value for predicting the clinical outcome during GMA therapy remains unclear. Measuring the FC concentration may allow for invasive procedures to be avoided if it can reasonably estimate the clinical outcome during GMA. Thus, the present study investigated the relationship between the clinical outcome and time-dependent change in the FC level during GMA and evaluated the utility of the FC concentration as a biomarker for predicting the clinical outcome during GMA in patients with active UC.

## Materials and methods

### Study design and ethics

This multicenter prospective observational study was conducted to assess the utility of FC as a biomarker for assessing the clinical efficacy and mucosal healing during GMA with an Adacolumn in patients with active UC by three independent institutes in Japan from October 2015 to March 2019. These institutes included Asahikawa Kosei General Hospital, Asahikawa City Hospital and Asahikawa Medical University. The protocol was reviewed and approved by the Ethics Committees at Asahikawa Medical University (approved number 15106) and each institution. All patients provided their written informed consent in accordance with the Declaration of Helsinki before commencement of this study.

### Patients

The inclusion criteria were as follows: (1) a diagnosis of UC, (2) ≥ 16 years of age; and (3) any clinical symptoms with a partial Mayo (pMayo) score of ≥ 3 (stool frequency [0–3], rectal bleeding [0–3] and Physician’s global assessment [0–3]). The exclusion criteria were as follows: (1) contraindication of GMA therapy (according to our hospital criteria: neutrophil count < 1500/mm^3^, hemoglobin < 10 g/dL or a history of allergic reaction to anticoagulant or a serious cardiac, pulmonary, hepatic or renal disorder), (2) total colectomy, and (3) taking > 2 oral NSAID tablets per week.

Patients receiving 5-aminosalicylic acid preparation, immunosuppressors, corticosteroids or biologic at entry were able to continue taking these medications at the same dose and frequency. Additional new medication for UC was not allowed during GMA. However, for patients receiving corticosteroids at entry, the dose could be reduced or treatment could be stopped according to improvement during GMA. When a patient’s condition worsened or remained unchanged, GMA could be withdrawn and another therapy including corticosteroids, immunosuppressants, biologics or surgery could be initiated, as necessary. The following parameters at entry were recorded in all patients as baseline characteristics: age, gender, body weight, history, duration of disease, extent of UC, current exacerbation, and concomitant medication.

### GMA therapy with an Adacolumn

GMA with an Adacolumn (JIMRO Co., Takasaki, Japan) is approved as remission induction therapy for patients with active UC by the Japan Ministry of Health. All patients in our study received 10 sessions of GMA with an Adacolumn twice a week for 5 consecutive weeks. Each patient’s blood via venipuncture of an antecubital vein entered the Adacolumn and then was delivered back to the patient via the column outflow line. The GMA regimen consisted of a standard protocol, filtering 1800 ml per session at a rate of 30 ml/min for 60 min. An optimum dose of sodium heparin (5000 units/session) was administered during GMA as an anticoagulant. All adverse events that occurred during GMA were recorded.

### Biomarker measurement

FC was measured at entry, 1 week, 2 weeks, at the end of GMA and on the day of endoscopy within 24 weeks after GMA. All patients were required to collect a stool sample within two days before their clinical visit and store it at room temperature. The stool samples were sent to Thermo Fisher Scientific (Tokyo, Japan) in a frozen state, and the calprotectin concentration was measured in a blinded manner to determine the clinical and endoscopic profiles. After thawing, the stool samples were homogenized by mixing with a predefined extraction buffer volume. After centrifugation, the supernatants were subjected to a fluorescence enzyme immunoassay using EliA Calprotectin 2. The measurements were performed in duplicate, and the average value was used for the analyses. Laboratory values, including the white blood cell (WBC) count and C reactive protein (CRP) level were also measured at the same time points as FC measurement: at entry, one and two weeks and at the end of GMA and on the day when endoscopy was performed within 24 weeks after GMA.

### The assessment of the clinical efficacy of GMA with Adaculumn

Clinical activity was assessed using the pMayo score at the same time points as FC was monitored: at entry, one week, two weeks, and at the end of GMA. Clinical remission (CR) was defined as a pMayo score of ≤ 2 and a score of ≤ 1 for all subscores (stool frequency [0–3], rectal bleeding [0–3] and Physician’s global assessment [0–3]). Endoscopy was performed before starting GMA and within 24 weeks after finishing GMA. The endoscopic activity was assessed using the Mayo endoscopic subscore (MES) at the most severely inflamed segment of the colon. Endoscopic remission (ER) which refers to mucosal healing, was defined as MES 0 or 1.

### Endpoints

The primary endpoint was the relationship between the ER and the FC concentration after GMA therapy. The secondary endpoints were the relationships between the CR and the time-dependent changes in the pMayo score, FC concentration, WBC count and CRP value during GMA therapy.

### Statistical analyses

The numerical data presented as the medians with interquartile ranges (IQRs). The comparison of demographic characteristics between the CR and non-CR groups was performed using the Mann–Whitney U-test or chi-square test. Wilcoxon’s test was used for nonparametric, paired and continuous variables, such as the time-dependent change in each marker during GMA. Receiver operating characteristics (ROC) curves with their area under the curve (AUC) were used to assess the cut-off value of endoscopic remission and predictors of CR with GMA therapy. The point with the largest AUC was defined as the point having the greatest association with predicting the CR with GMA therapy. The optimum cut-off value was determined as the nearest point from upper left corner to the point on the ROC curve. A *P* value < 0.05 was considered statistically significant. Every statistical analysis was performed using the SPSS for Windows (SPSS Inc., Chicago, IL, USA).

## Results

### Patients’ characteristics

A total 36 of patients with active UC were enrolled in this study. Ten patients were excluded (administration of additional medication during GMA therapy [n = 5]; issues with laboratory results [n = 2]; deviation from protocol [n = 2]; total colectomy [n = 1]); thus, 26 patients were included in the study. The baseline characteristics of the 26 eligible patients in this study are shown in Table 1.

**Table 1:** The baseline characteristics of the 26 eligible patients in this study

Baseline characteristics	N	26	
Age	Median (min–max)	39 (16–70)	
Gender—male	N (%)	11(42.3)	
Body weight, kg	Median (min–max)	51.9 (37.7–87.7)	
Duration of disease, month	Median (min–max)	58 (10–159)	
Extent of UC (Montreal classification)			
	Left side colitis (E2)	N	10
	Pancolitis (E3)	N	16
Disease activity			
	pMayo score	Median (IQR)	6 (5.25–7)
	MES	Median (IQR)	2 (2–2)
Concomitant medication			
	5-aminosalicylic acid	N (%)	23 (88.4)
	Corticosteroid	N (%)	8 (30.7)
	Immunosuppressant	N (%)	11 (42.3)
	Anti-TNF-alpha agent	N (%)	5 (19.2)
Biomarker			
	FC (mg/kg)	Median (IQR)	11,294.5 (5116.75–18,755.25)
	WBC count	Median (IQR)	6350 (4935–7945)
	CRP (mg/dL)	Median (IQR)	0.58 (0.1–1.39)

### The overall efficacy and safety of GMA with an Adacolumn and the baseline characteristics of CR patients.

According to the clinical assessment, 13 patients achieved a CR and 13 did not achieve a CR at the end of treatment. According to the endoscopic assessment, among the 13 patients with a CR, 5 achieved an ER after GMA therapy. The overall CR and ER rates were 50.0% and 19.2%, respectively. There were no adverse events in the present study.

The patients were divided into the CR and non-CR groups by the assessment of the clinical activity based on the pMayo score at the end of GMA. The baseline characteristics of the groups at the start of GMA are shown in Table 2. There were no significant differences in age, gender, body weight or extent of UC. However, the disease duration was significantly longer in the CR group than in the non-CR group: 118 vs. 28 months (*P* = 0.001). There was no significant difference in the pMayo score between the CR and non-CR groups, although the MES at the start of GMA in the CR group was significantly lower than that in the non-CR group: 2 vs. 2 (*P* = 0.03). There was no significant difference in the ratio of concomitant medication, such as 5-aminosalicylic acid, corticosteroids, immunosuppressants and anti-TNF-α agents, or in the biomarkers of FC, WBC count and CRP at the start of GMA between the CR and non-CR groups.

**Table 2:** The comparison of baseline characteristics between the CR and non-CR groups

Baseline characteristics			CR group	Non-CR group	*p* value
Number of cases		N	13	13	
Age		Median (min–max)	53 (25–68)	23 (16–70)	0.09
Gender—male		N (%)	4	7	0.234
Body weight, kg		Median (min–max)	47 (43.7–68.2)	55 (37.7–83.3)	0.48
Duration of disease, month		Median (min–max)	118 (12–159)	28 (6–130)	0.001
Extent of UC (Montreal classification)					0.42
	left side colitis (E2)	N	4	6	
	pancolitis (E3)	N	9	7	
Disease activity					
	pMayo score	Median (IQR)	6 (6–7)	7 (5–7)	0.76
	MES	Median (IQR)	2 (2–2)	2 (2–3)	0.03
Concomitant medication					
	5-aminosalicylic acid	N (%)	12	10	
	Corticosteroid	N (%)	4	3	0.658
	Immunosuppressant	N (%)	5	8	0.239
	Anti-TNF-alpha agent	N (%)	3	2	0.619
Biomarker at entry					
	FC (mg/kg)	Median (IQR)	15,669 (7280–20,017)	9496 (1073–14,015)	0.07
	WBC count	Median (IQR)	5980 (4760–6970)	7290 (5200–8250)	0.29
	CRP (mg/dL)	Median (IQR)	0.66 (0.1–4.56)	0.36 (0.1–1.26)	0.53

**Table 3:** The cut-off value of each marker and the sensitivity, specificity, positive predictive value and accuracy

	Sensitivity	Specificity	PPV	Accuracy
pMayo≤4	0.615	0.923	0.888	0.769
pMayo≤5	0.769	0.846	0.833	0.807
pMayo≤6	0.846	0.461	0.611	0.653
FC≤4500	0.615	0.615	0.615	0.615
FC≤5000	0.692	0.615	0.642	0.653
FC≤5500	0.692	0.538	0.6	0.615
CRP≤0.4	0.769	0.461	0.588	0.615
CRP≤0.6	0.923	0.461	0.631	0.692
CRP≤0.8	0.923	0.384	0.6	0.653
ΔFC≤30	0.692	0.846	0.818	0.769
ΔFC≤40	0.769	0.846	0.833	0.807
ΔFC≤50	0.769	0.969	0.769	0.769
ΔCRP≤60	0.615	0.846	0.8	0.73
ΔCRP≤70	0.666	0.846	0.8	0.76
ΔCRP≤80	0.666	0.769	0.727	0.72

### Primary endpoint: Relationship between ER and the FC level after GMA therapy

**Fig. 1:**
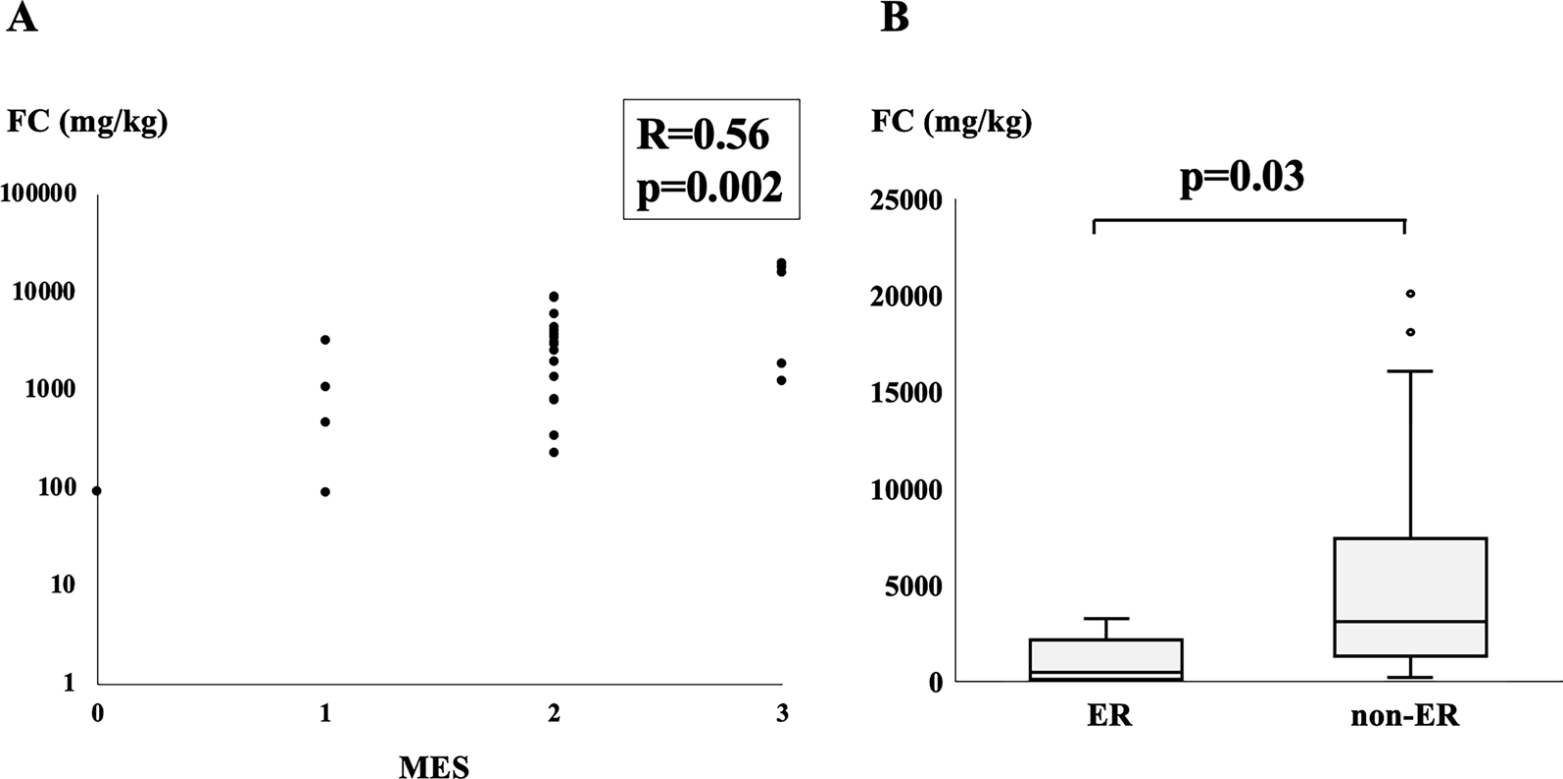
The relationship between the ER and the FC concentration after GMA. The FC concentration after GMA was well correlated with the MES (R = 0.56, *p* = 0.002) (**a**). The median FC concentration of the ER group was significantly lower than that of the non-ER group after GMA (469 mg/kg vs. 3107 mg/kg, p = 0.03) (**b**). ER: endoscopic remission, FC: fecal calprotectin, MES: Mayo endoscopic score, GMA: granulocyte and monocyte adsorptive apheresis

The FC concentration after GMA was well correlated with the MES (R = 0.56, *p* = 0.002) (Fig. 1a). The 26 patients were divided into the ER (n = 5) and a non-ER (n = 21) group. The median FC concentration of the ER group was significantly lower than that of the non-ER group after GMA (469 mg/kg vs. 3107 mg/kg, *p* = 0.03) (Fig. 1b). An ROC analysis showed that when the cut-off value of FC concentration was set at 1150 mg/kg for assessing ER after GMA, the sensitivity, specificity, positive predictive value (PPV) and accuracy were 0.8, 0.81, 0.5 and 0.8, respectively.

### Secondary endpoint: Relationship between CR and time-dependent changes in the pMayo score, FC level, WBC count and CRP during GMA therapy

**Fig. 2:**
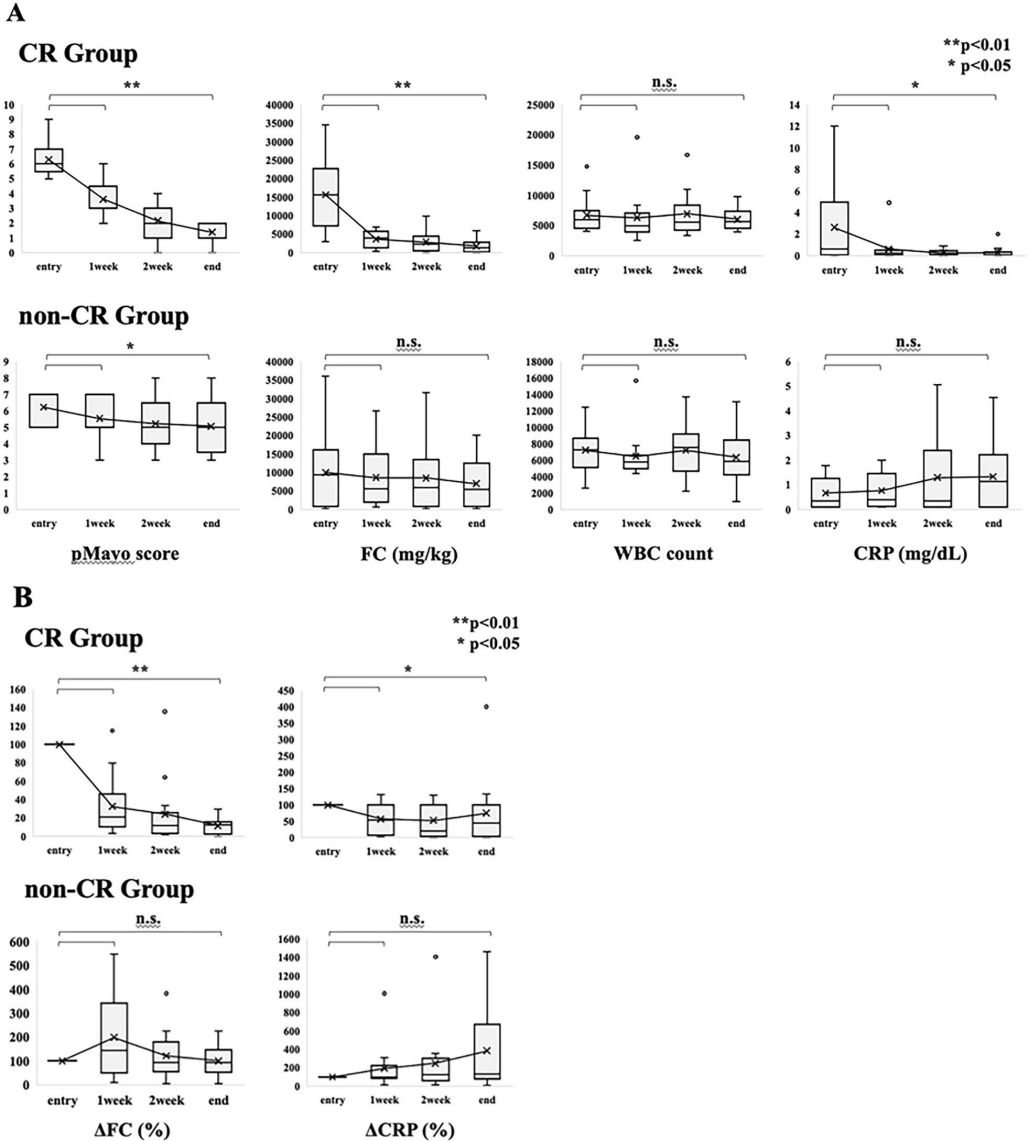
Time-dependent changes in pMayo score, FC, WBC, CR, ΔFC and ΔCRP during GMA. A time-dependent decline in the median pMayo score during GMA was observed in both the CR and non-CR groups. A time-dependent decrease in the median FC concentration during GMA was only observed in the CR group. A time-dependent change in the WBC count was not observed during GMA in either the CR or non-CR groups. A time-dependent decrease in the median CRP value during GMA was only observed in the CR group (**a**). Time-dependent decreases in the median ΔFC and median ΔCRP during GMA were only observed the CR group (**b**). The pMayo score, FC concentration, CRP value, ΔFC and ΔCRP at one week showed remarkable reductions from their values at entry (**a**, **b**). * and ** indicate statistically significant differences in comparison to baseline; *p* < 0.05 and *p* < 0.01, respectively. pMayo: partial Mayo score, FC: fecal calprotectin, WBC: white blood cell, CRP: C reactive protein, ΔFC: the reduction rate of FC, ΔCRP: the reduction rate of CRP, GMA: granulocyte and monocyte adsorptive apheresis, CR: clinical remission

The time-dependent changes in the pMayo score, FC concentration, WBC count and CRP value during GMA are shown in Fig. 2. A time-dependent decline in the pMayo score during GMA was observed in both the CR and non-CR groups. In particular, the pMayo score at 1 week was significantly lower than at entry in both the CR and non-CR groups (*p* < 0.01, *p* < 0.05). A time-dependent decrease in the FC concentration during GMA was only observed in the CR group, and the FC concentration at 1 week was significantly lower than that at entry (*p* < 0.01). A time-dependent change in the WBC count was not observed during GMA in either the CR or non-CR groups. A time-dependent decrease in the CRP value during GMA was only observed in the CR group, and the CRP value at 1 week was significantly lower than that at entry (*p* < 0.05) (Fig. 2a).

Both the FC concentration and CRP value at entry showed a wide range of measurements. FC is known to have wide variability, which is influenced by various factors [6]. CRP is also affected by bacterial infections in the internal and external intestine. We therefore also analyzed the reduction rates of FC (ΔFC) and CRP (ΔCRP) to assess the accuracy of these biomarkers in predicting the efficacy of GMA. Similar to findings for the FC concentration and CRP value, time-dependent decreases in ΔFC and ΔCRP during GMA were only observed in the CR group, and the ΔFC and ΔCRP values at 1 week were significantly lower than at entry (*p* < 0.01, 0.05) (Fig. 2b). The pMayo score, FC concentration, CRP value, ΔFC and ΔCRP at one week showed remarkable reductions from their values at entry; thus, they were considered potential predictors of CR at the end of GMA.

**Fig. 3:**
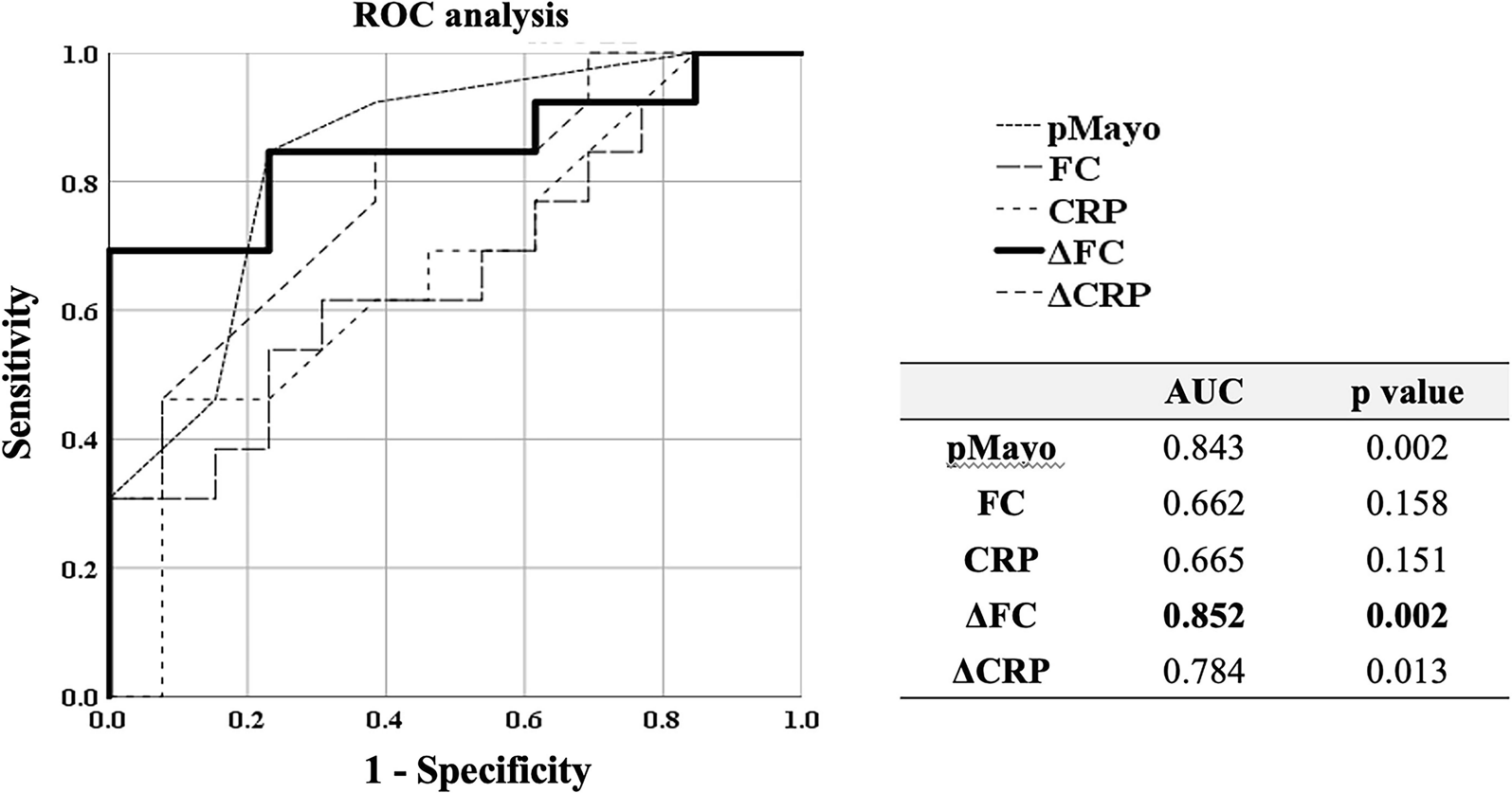
Comparison of each marker as a predictor of CR at the end of GMA therapy. The ΔFC value at week 1 was demonstrated the most accurate predictor of CR at the end of GMA (AUC = 0.852, *p* = 0.002) among the pMayo score, FC concentration, CRP value and ΔCRP. (AUC = 0.842, 0.663, 0.666 and 0.784. *p* = 0.003, 0.158, 0.151 and 0.014) CR: clinical remission, GMA: granulocyte and monocyte adsorptive apheresis, ΔFC: the reduction rate of fecal calprotectin, pMayo: partial Mayo score, FC: fecal calprotectin, CRP: C reactive protein, ΔCRP: the reduction rate of CRP

An ROC analysis showed that ΔFC at 1 week was the most accurate predictor of CR at the end of GMA (AUC = 0.852, *p* = 0.002) among the pMayo score, FC concentration, CRP value and ΔCRP at 1 week (AUC = 0.842, 0.663, 0.666 and 0.784. P = 0.003, 0.158, 0.151 and 0.014) (Fig. 3). The cut-off value of each marker and the sensitivity, specificity, PPV and accuracy are shown in Table 3. When the cut-off value of ΔFC at 1 week for predicting CR at the end of GMA therapy was set at ≤ 40%, the sensitivity, specificity, PPV and accuracy were 76.9%, 84.6%, 83.3% and 80.7%, respectively (Table 3).

## Discussion

To our knowledge, this is the first study to evaluate the utility of FC as a biomarker for predicting the clinical outcome during GMA in patients with active UC. In particular, FC showed efficacy as a biomarker in two respects: it was useful for predicting not only ER after GMA but also CR at the early phase during GMA.

Regarding the relationship between ER and the FC concentration after GMA, our results demonstrated that the FC concentration after GMA was able to assess ER and the therapeutic outcome of GMA without the need for any endoscopic procedure. Shimoyama et al. also reported that the FC level showed a positive relationship with the MES and could be used to assess the endoscopic disease activity at the end of GMA [23]. In their study, although the cut-off value for assessing ER was not calculated, the median FC concentration in patients with mucosal healing was significantly lower than that in those with non-mucosal healing, which was consistent with our finding that the FC concentration after GMA was able to predict ER. Although the baseline characteristics and disease activity in their study were not markedly different from our study, the median FC concentration at entry in their study was markedly lower than that in our study. Labaere et al. reported that large quantitative differences of FC concentration were observed among various assays used in clinical practice [29]. This discrepancy regarding FC concentrations between the present study and the study of Shimoyama et al. may be due to differences in measurement assays. The further standardization of FC measurement is needed in order to establish a unified cut-off value for assessing ER.

We also investigated the relationship between CR and time-dependent changes in the pMayo score, FC concentration, WBC count and CRP value during GMA. According to an ROC analysis, ΔFC at 1 week was the most accurate predictor of CR at the end of GMA. In the present study, a pMayo score cut-off value of 5 showed the same result as a ΔFC cut-off value of ≤ 40% at 1 week (Table 3). However, the pMayo score is a subjective marker, as it includes the Physician’s global assessment, which is performed by each physician. One advantage of the pMayo score is that it is simple to determine; however, the physician’s experience is extremely influential on the judgment of the general condition. Thus, the ΔFC at 1 week from starting GMA was shown to be the most accurate and objective predictor of the clinical outcome after GMA.

Previous reports have shown that patients who were most likely to respond to GMA tended to be first-episode cases who were steroid-naïve with a short disease duration and low disease activity [21–23]. However, in our study, the disease duration in the CR group was significantly longer than that in the non-CR group, a finding that is completely opposite those of previous clinical trials. Furthermore, the rates of concomitant medication, such as corticosteroids, immunosuppressants and anti-TNF-α agents, did not differ between the CR and non-CR groups to a statistically significant extent. Thus, we suspect that assessing only the baseline characteristics before GMA therapy is not sufficient to predict CR, and a useful objective biomarker is important for evaluating the clinical efficacy of GMA.

Toyonaga et al. reported that FC measurement failed to predict the outcomes of remission induction therapy, including corticosteroids, anti-TNF-α agents and calcineurin inhibitors, in patients with active UC because of wide within-day variability [12]. Turner et al. reported that FC levels in pediatric patients were unchanged at three days after intravenous steroid therapy [13]. In contrast, De Vos et al. reported that the FC concentration at two weeks after remission induction therapy with infliximab was significantly lower in patients who achieved endoscopic remission than in those that did not [16]. The mechanism underlying the effects of GMA with an Adacolumn involves the selective removal of cell populations, including granulocytes, monocytes and macrophages, from the peripheral blood [17, 18]. GMA is reported to decrease the mucosal level of neutrophils [30] and the peripheral blood level of CD14(+)CD16(+) proinflammatory monocytes while concomitantly increasing the numbers of immature monocytes [31, 32]. GMA would therefore decrease the number of neutrophils and macrophages in the intestinal mucosa via the Adacolumn, which adsorbs granulocytes and monocytes from the peripheral blood. In addition, the FC concentration is reportedly strongly correlated with the number of neutrophils in stool samples in patients with IBD [33]. The early decrease in the FC concentration immediately after GMA in the present study might be a specific reaction associated with GMA.

The strength of the present study is its prospective design and the fact that it was conducted among multiple facilities, all of which employed the same protocol. However, it was associated with some limitations. Specifically, the study population was relatively small and we did not conduct histological assessments. Further investigations in larger cohorts will be needed to confirm the utility of FC as a biomarker.

## Conclusion

In summary, we demonstrated the utility of FC as a biomarker for assessing ER after GMA and predicting CR at the early phase during GMA in patients with active UC. Assessing the baseline characteristics alone before GMA was not sufficient to predict CR. Our findings will benefit patients with active UC by allowing them to avoid undergoing unnecessary invasive procedures and will help establish new GMA therapeutic strategies.

## Data Availability

All data generated or analyzed during this study are included in this published article.

## Abbreviations

UC: Ulcerative colitis
IBD: Inflammatory bowel diseases
FC: Fecal calprotectin
GMA: Granulocyte and monocyte adsorptive apheresis
FcγR: Fragment crystallizable-gamma receptor
WBC: White blood cell
CRP: C reactive protein
pMayp: Partial Mayo
MES: Mayo endoscopic subscore
CR: Clinical remission
non-CR: Non-clinical remission
ER: Endoscopic remission
non-ER: Non-endoscopic remission
IQRs: Interquartile ranges
ROC: Receiver operating characteristics
AUC: Area under the curve
PPV: Positive predictive value
ΔFC: Reduction rate of FC
ΔCRP: Reduction rate of CRP
